# Pulmonary Nocardiosis in an Immunocompetent Host: A Diagnostic Challenge Requiring Multimodal Evaluation

**DOI:** 10.7759/cureus.98695

**Published:** 2025-12-08

**Authors:** Vikram Oke, Seerut Dhillon, Michelle Ju, Robert Tilley, Karthik Iyer

**Affiliations:** 1 Pulmonary and Critical Care Medicine, Mercy Hospital Jefferson, Festus, USA; 2 Internal Medicine, Mercy Hospital Jefferson, Festus, USA; 3 Critical Care Medicine, Mercy Hospital Jefferson, Festus, USA

**Keywords:** bronchoalveolar lavage, endobronchial ultrasound-guided transbronchial needle aspiration (ebus-tbna), immunocompetent, multimodal diagnosis, nocardia cyriacigeorgica, opportunistic infection, pulmonary fibrosis, pulmonary nocardiosis

## Abstract

Pulmonary nocardiosis is a rare opportunistic infection usually affecting immunocompromised hosts but can present in immunocompetent individuals with chronic lung disease, creating diagnostic challenges. We describe the case of a 72-year-old woman with pulmonary fibrosis who had progressive pneumonia unresponsive to standard therapy. Despite multiple non-diagnostic bronchoscopies, cultures from an endobronchial ultrasound-guided transbronchial needle aspiration of a mediastinal lymph node ultimately confirmed *Nocardia *species. This case underscores the importance of considering nocardiosis in non-resolving pneumonia among immunocompetent patients and highlights the role of multimodal, invasive diagnostics in establishing a timely diagnosis and guiding targeted therapy.

## Introduction

Pulmonary nocardiosis is a rare but potentially serious opportunistic infection caused by *Nocardia* species.* Nocardia *spp. are aerobic, filamentous, partially acid-fast, gram-positive bacteria found primarily within soil, decaying organic matter, and water [1,2]. The most common form of nocardiosis is associated with pulmonary involvement, typically resulting from inhalation of the bacteria [3]. Nocardiosis most commonly affects immunocompromised individuals, including those with malignancies, solid organ transplant recipients, on chronic corticosteroid therapy, or those with a history of human immunodeficiency virus (HIV) or acquired immune deficiency syndrome (AIDS) [1,4]. Over one-third of these infections occur in immunocompetent hosts, particularly in chronic lung diseases such as chronic obstructive pulmonary disease or bronchiectasis [3,5]. Pulmonary nocardiosis is difficult to diagnose as symptoms are subacute and overlap with chronic lung disease exacerbations and other infections. The diagnosis requires a high index of suspicion, especially in immunocompetent patients. We present a diagnostically challenging case of pulmonary nocardiosis in an immunocompetent patient with pulmonary fibrosis. The diagnosis required multiple invasive diagnostic modalities, including repeat bronchoscopies, with endobronchial ultrasound-guided transbronchial needle aspiration (EBUS-TBNA) of a mediastinal lymph node ultimately providing confirmation.

## Case presentation

A 72-year-old woman with a history significant for pulmonary fibrosis and chronic kidney disease (CKD) presented to the emergency department (ED) with bilateral pleuritic chest pain, productive cough, fevers, diarrhea, and generalized weakness. She had a 10-pack-year smoking history but quit 40 years prior. Five days before presentation, her primary care physician empirically treated her with azithromycin for presumed community-acquired pneumonia, but her symptoms worsened, prompting ED referral. A review of her medication list confirmed she was not taking any immunosuppressive medications.

**Figure 1:**
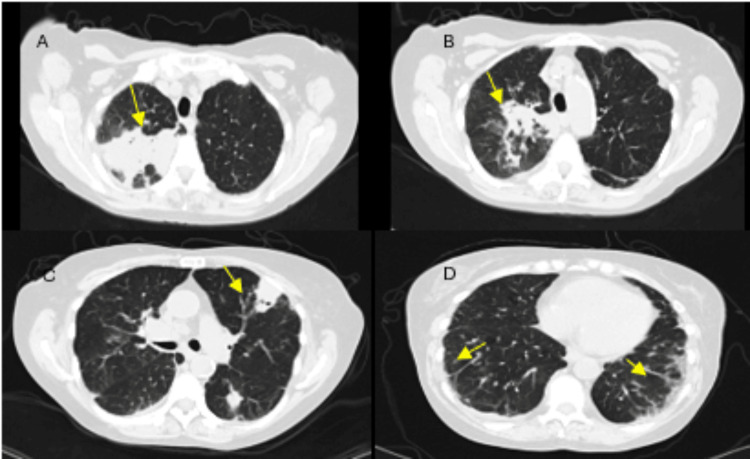
CT of the chest without contrast showing multifocal pulmonary opacities (arrows) in the right upper lobe (A, B), left upper lobe (C), and fibrotic changes at the lung bases (D). The right and left upper lobe opacities contain several bubbles of air, which may indicate a cavitary process.

On admission, the patient was tachycardic with a heart rate of 112 beats/minute but otherwise hemodynamically stable. Physical examination revealed bilateral basilar crackles. Laboratory studies were significant for leukocytosis (white blood cell count = 16.6 K/µL, normal range = 4-11 K/µL), mild anemia (hemoglobin = 11.6 g/dL, normal range = 11.9-15 g/dL), hyponatremia (Na = 130 mmol/L, normal range = 136-145 mmol/L), and elevated blood urea nitrogen (42 mg/dL, normal range = 8-23 mg/dL) and creatinine (1.37 mg/dL, normal range = 0.51-0.95 mg/dL). Serum lactate was not elevated. CT of the chest without contrast demonstrated multifocal pulmonary opacities with airspace disease, cavitary changes bilaterally (Figure 1).

A preliminary diagnosis of Gram-negative pneumonia was made, and the patient was started on cefepime and metronidazole; however, despite the antimicrobial therapy, she continued to have fevers and progressive infiltrates. Due to a history of chicken coop exposure and episodes of dysphagia, aspiration pneumonia and zoonotic infections were considered. This workup included obtaining blood and sputum cultures, serum and urine fungal antigens, and serologies for *Bartonella*, Q fever, and *Toxoplasma*, all of which were negative. The QuantiFERON test was negative as well (Table 1).

**Table 1:** Summary of pertinent laboratory values during hospitalization. *: Panel includes adenovirus; coronavirus HKU1, NL63, 229E, and OC43; influenza A H1, H1-2009, and H3; influenza B; human metapneumovirus; parainfluenza virus 1, 2, 3, and 4; respiratory syncytial virus; rhinovirus/enterovirus; *Bordetella pertussis*; *Chlamydophila pneumoniae*; and *Mycoplasma pneumoniae*. WBC = white blood cells; PCR = polymerase chain reaction; ANA = antinuclear antibodies; BAL = bronchoalveolar lavage; EBUS-TBNA = endobronchial ultrasound-guided transbronchial needle aspiration

Test	Result
Sputum anaerobic/aerobic culture with Gram stain	No aerobic or anaerobic growth; no organisms or WBCs observed
Sputum fungal culture	No fungus isolated
Blood culture	No growth on days 1 and 5
*Legionella* antigen, urine	Not detected
*Streptococcus pneumoniae* antigen, urine	Not detected
QuantiFERON TB Gold Plus	Negative
Respiratory pathogen PCR panel*	No respiratory pathogen nucleic acids detected
*Histoplasma* antigen, urine	Negative
Cryptococcal antigen, blood	Negative
*Bartonella henselae* IgG titers	<1:128
*Bartonella henselae* IgM titers	<1:20
*Coxiella burnetii* antibodies	None detected
Fungal culture, sputum	1+ or few *Candida albicans*; scant growth *Candida krusei*
Fungal culture, blood	No growth
*Toxoplasmosis* IgG and IgM, blood	Negative
ANA screen	Negative
Rheumatoid factor	<10 (reference range <14 IU/mL)
Cyclic citrullinated peptide antibody IgG	12 (reference range <17 U/mL)
Hepatitis A, B, C	Non-reactive
HIV-1 and 2 antibodies and HIV-1 antigen	Non-reactive
BAL right upper lobe: respiratory culture with Gram stain	<10,000 cfu/mL. Normal upper respiratory flora; no organisms observed, intracellular organisms absent, 2+ (few) WBC
BAL right upper lobe: fungal culture	Scant growth of *Candida albicans* and *Candida glabrata*
BAL right upper lobe: acid-fast bacilli culture with stain	No acid-fast bacilli isolated or observed
BAL left upper lobe: respiratory culture with Gram stain	<10,000 cfu/mL. Normal upper respiratory flora; non-diagnostic pattern, 3+ (moderate) WBC, intracellular organisms absent
BAL left upper lobe: fungal culture	Scant growth of *Candida albicans* and *Candida glabrata*
BAL left upper lobe: acid-fast bacilli culture with stain	No acid-fast bacilli isolated or observed
EBUS-TBNA: anaerobic/aerobic culture with Gram stain	No aerobic or anaerobic growth; no organisms or WBC observed
EBUS-TBNA: fungal culture	No fungus isolated
EBUS-TBNA: acid-fast bacilli culture with stain	No acid-fast bacilli observed on acid-fast bacilli stain or culture; Grocott’s methenamine silver stain with rare branching, filamentous structures, possibly representing fungal hyphae; acid-fast bacilli culture positive for *Nocardia* species
EBUS-TBNA: cytology	Necrotic cellular debris and neutrophils

**Figure 2:**
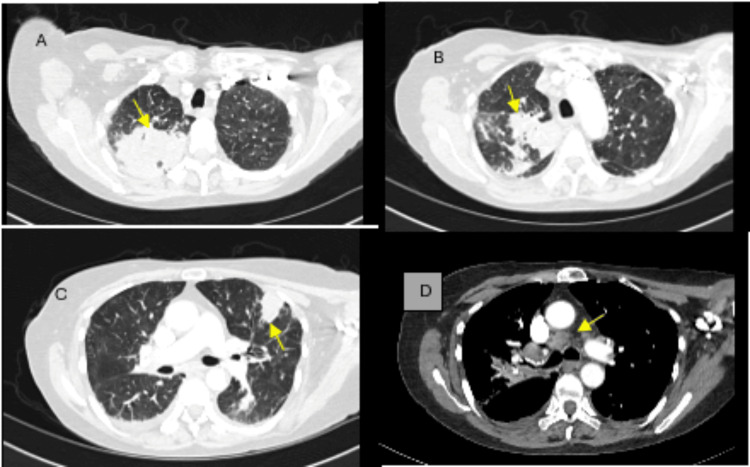
Repeat CT of the chest with contrast after treatment with multiple broad-spectrum antibiotics showing worsening multilevel lung infiltrates (A-C arrows). The mediastinal window demonstrates an enlarged right paratracheal lymph node (D, arrows).

**Figure 3:**
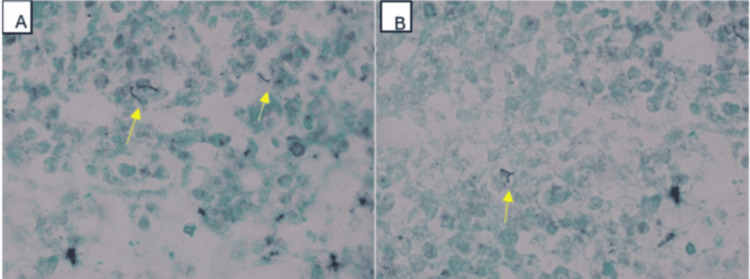
Grocott’s methenamine silver stain of the lymph node aspirate showing rare branching filamentous structures (arrows, in A and B), confirmed by culture to be Nocardia cyriacigeorgica.

A pulmonologist performed a bronchoscopy with bronchoalveolar lavage (BAL). No endobronchial lesions were visualized during the procedure. BAL fluid cultures were initially unremarkable, growing scant *Candida* species, which were considered to be colonization. Acid-fast bacilli (AFB) stains and cultures were negative, and cytology did not reveal atypical cells. Despite multiple broad-spectrum antibiotics, the patient continued to experience persistent fevers. A repeat chest CT revealed worsening pulmonary consolidations and an enlarged right paratracheal lymph node, prompting a second bronchoscopy with BAL and EBUS-TBNA of the paratracheal lymph node (Figure 2). Results were similar to prior except cytology from the lymph node aspirate revealed acute inflammation and necrosis. The histochemical stains were negative for AFB (Table 1). Grocott’s methenamine silver stain identified rare filamentous structures, which were thought to represent fungal hyphae (Figure 3). As part of the ongoing evaluation for potential underlying immunosuppression or systemic diseases, she was also tested for HIV, hepatitis C, and antinuclear antibodies; all results were negative (Table 1).

**Figure 4:**
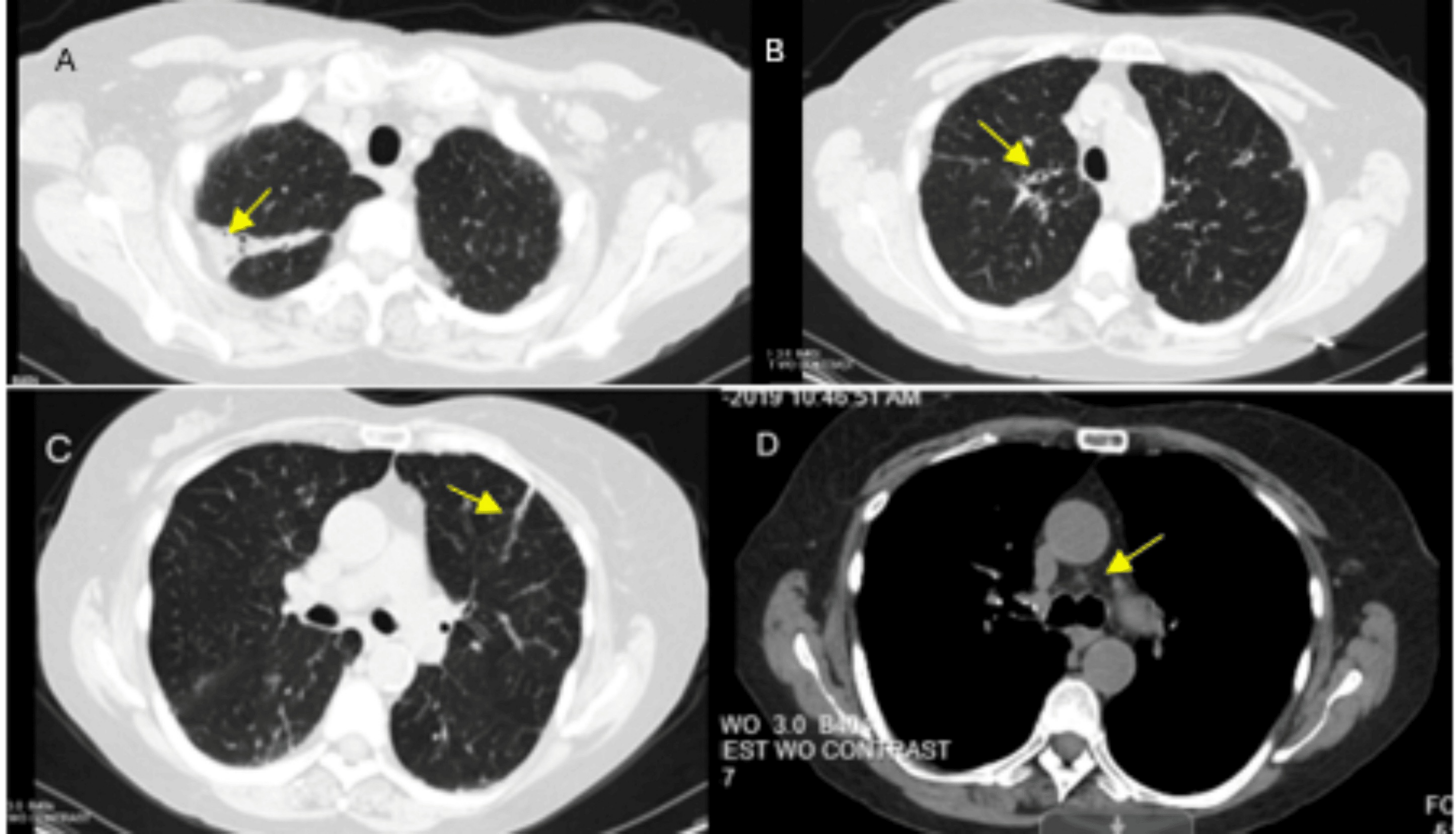
CT of the chest without contrast showing significant improvement in previously noted areas of consolidation and mediastinal lymphadenopathy (A-C). The mediastinal window reveals significantly improved right paratracheal lymphadenopathy (D).

In view of clinical and radiologic deterioration despite adequate antibiotic therapy, an unremarkable infectious workup, cryptogenic organizing pneumonia was suspected. Prednisone 60 mg daily was initiated before discharge with a planned pulmonary follow-up. Several days later, the lymph node cultures from the EBUS-TBNA grew *Nocardia cyriacigeorgica*, prompting initiation of trimethoprim-sulfamethoxazole for treatment of pulmonary nocardiosis. Prednisone was tapered and discontinued. The patient developed a maculopapular rash with trimethoprim-sulfamethoxazole, necessitating a transition to minocycline for 12 months. At pulmonary follow-up, the patient reported continued improvement, and six-month CT imaging demonstrated improvement in bilateral opacities and mediastinal adenopathy (Figure 4).

## Discussion

Pulmonary nocardiosis is an uncommon but serious infection caused by *Nocardia*, an aerobic filamentous Gram-positive bacterium found in soil and water [1,2]. According to the Centers for Disease Control and Prevention, more than 40 species are considered clinically relevant, with* Nocardia nova*,* Nocardia farcinica*,* Nocardia cyriacigeorgica*,* Nocardia brasiliensis*, and *Nocardia abscessus* most frequently isolated. Among these, *Nocardia cyriacigeorgica* is reported as the most prevalent cause of human nocardiosis in North America [6,7]. Infections occur predominantly in immunocompromised hosts, including solid organ or bone marrow transplants, HIV/AIDS, and chronic corticosteroid use [1,4,6,8]. A review of 35 case reports of *Nocardia cyriacigeorgica* showed 58% of patients were immunocompromised, highlighting the higher prevalence of this species in immunocompromised compared with immunocompetent hosts [9]. The incidence overall appears to be increasing, likely reflecting improved survival among immunocompromised populations and the broader use of immunosuppressive therapies [10].

Clinically, pulmonary nocardiosis follows a subacute course that evolves over several weeks. Systemic symptoms such as fever or leukocytosis may occur initially, but localized pulmonary symptoms such as cough, pleuritic chest pain, dyspnea, and hemoptysis become more prominent as the disease progresses [1,3,11]. As these features overlap with chronic lung disease exacerbations and other infections, diagnosis is often delayed. Suspicion should be heightened in patients with new pulmonary nodules, particularly in the context of chronic lung disease or prolonged corticosteroid use. Delay in establishing the diagnosis of pulmonary nocardiosis carries an increased mortality rate of 38% [12,13].

Diagnosis remains challenging and is often delayed despite extensive evaluation [4,12-14]. The gold standard is culture, but recovery requires specialized methods, such as fluorescent auramine-rhodamine, Kinyoun stains, or a selective medium, such as modified Thayer Martin agar, to facilitate growth. *Nocardia* can also grow on Lowenstein-Jensen media used for mycobacteria [12]. Additionally, non-invasive respiratory tract sampling demonstrated a higher diagnostic yield of 77% compared to invasive methods, such as bronchoscopy with BAL, at 47%. The mean interval from presentation to diagnosis was 42 days [4]. In our patient,* Nocardia cyriacigeorgica* was diagnosed via EBUS-TBNA of an enlarged paratracheal lymph node after two bronchoscopies and other studies were non-diagnostic, highlighting the diagnostic challenges of nocardiosis. The mean interval from presentation to diagnosis of pulmonary nocardiosis was 31 days in our patient.

Trimethoprim-sulfamethoxazole is the first-line treatment for pulmonary nocardiosis, extended to 12 months in severe disease. Due to possible resistance, severe infections warrant empiric therapy with two to three agents. Alternatives for sulfonamide intolerance include minocycline, dapsone, and third-generation cephalosporins [15].

## Conclusions

A 72-year-old female with underlying chronic fibrotic lung disease and CKD developed progressive pneumonia unresponsive to broad-spectrum antibiotics. Despite multiple non-diagnostic bronchoscopies with bronchoalveolar lavage, EBUS-TBNA ultimately yielded *Nocardia*, confirming the diagnosis of pulmonary nocardiosis. Targeted therapy led to clinical recovery. This case report highlights the diagnostic challenge of pulmonary nocardiosis in an immunocompetent patient. Multiple non-invasive and invasive evaluations were required, with diagnosis ultimately confirmed by EBUS-TBNA. This case underscores the importance of considering pulmonary nocardiosis even in the absence of classic risk factors and emphasizes the value of a multimodal diagnostic approach for timely identification.

## References

[1] Wilson JW (2012). Nocardiosis: updates and clinical overview. Mayo Clin Proc.

[2] Brown-Elliott BA, Brown JM, Conville PS, Wallace RJ Jr (2006). Clinical and laboratory features of the Nocardia spp. based on current molecular taxonomy. Clin Microbiol Rev.

[3] Lerner PI (1996). Nocardiosis. Clin Infect Dis.

[4] Martínez Tomás R, Menéndez Villanueva R, Reyes Calzada S, Santos Durantez M, Vallés Tarazona JM, Modesto Alapont M, Gobernado Serrano M (2007). Pulmonary nocardiosis: risk factors and outcomes. Respirology.

[5] Beaman BL, Beaman L (1994). Nocardia species: host-parasite relationships. Clin Microbiol Rev.

[6] (2025). Centers for Disease Control and Prevention. Clinical overview of nocardiosis. https://www.cdc.gov/nocardiosis/hcp/clinical-overview/.

[7] Yang C, Zheng YX, Gu HY (2025). Genomic characteristics, virulence potential, antimicrobial resistance profiles, and phylogenetic insights into Nocardia cyriacigeorgica. Ann Clin Microbiol Antimicrob.

[8] Gabay S, Yakubovsky M, Ben-Ami R, Grossman R (2022). Nocardia cyriacigeorgica brain abscess in a patient on low dose steroids: a case report and review of the literature. BMC Infect Dis.

[9] Zuo H, Ye J, Li C (2024). Myasthenia gravis complicated with pulmonary infection by Nocardia cyriacigeorgica: a case report and literature review. Front Med (Lausanne).

[10] Fatahi-Bafghi M (2018). Nocardiosis from 1888 to 2017. Microb Pathog.

[11] Duggal SD, Chugh TD (2020). Nocardiosis: a neglected disease. Med Princ Pract.

[12] Murray PR, Heeren RL, Niles AC (1987). Effect of decontamination procedures on recovery of Nocardia spp. J Clin Microbiol.

[13] González-Jiménez P, Méndez R, Latorre A (2022). Pulmonary nocardiosis. A case report. Rev Esp Quimioter.

[14] Rathish B, Zito PM (2023). Nocardia. StatPearls.

[15] Restrepo A, Clark NM (2019). Nocardia infections in solid organ transplantation: guidelines from the Infectious Diseases Community of Practice of the American Society of Transplantation. Clin Transplant.

